# Advanced Endoscopic Imaging for Diagnosis of Crohn's Disease

**DOI:** 10.1155/2012/301541

**Published:** 2011-11-24

**Authors:** Helmut Neumann, Klaus Mönkemüller, Claudia Günther, Raja Atreya, Michael Vieth, Markus F. Neurath

**Affiliations:** ^1^Department of Medicine I, University of Erlangen-Nuremberg, 91054 Erlangen, Germany; ^2^Department of Internal Medicine, Gastroenterology and Infectious Diseases, Marienhospital Bottrop gGmbH, 46236 Bottrop, Germany; ^3^Institute of Pathology, Klinikum Bayreuth, 95445 Bayreuth, Germany

## Abstract

Endoscopy in IBD has tremendous importance to diagnose inflammatory activity, to evaluate therapeutic success and for the surveillance of colitis associated cancer. Thus it becomes obvious that there is a need for new and more advanced endoscopic imaging techniques for better characterization of mucosal inflammation and early neoplasia detection in IBD. This paper describes the concept of advanced endoscopic imaging for the diagnosis and characterization of Crohn's disease, including magnification endoscopy, chromoendoscopy, balloon-assisted enteroscopy, capsule endoscopy, confocal laser endomicroscopy, and endocytoscopy.

## 1. Introduction

Inflammatory bowel disease (IBD) comprises two major entities, ulcerative colitis (UC) and Crohn's disease (CD). It is now well accepted that both patient groups are at an increased risk of developing colitis-associated cancer (CAC) [1]. Chronic inflammation of the gastrointestinal mucosa represents an important factor for the development of colitis-associated cancer (CAC). Thus, chronic administration of anti-inflammatory drugs has been proposed to lower the risk for CAC. However, most studies could not demonstrate that commonly used anti-inflammatory drugs for treatment of IBD have chemopreventive effects against cancer [2]. Therefore, new attempts have been made to better characterize the development of mucosal inflammation in IBD in order to establish more effective preventive and targeted therapies. Indeed, very recently it was shown by Günther and coworkers that the cystein protease caspase 8 is critically involved in regulating intestinal homeostasis and in protecting intestinal epithelial cells from TNF-*α*-induced necroptotic cell death thereby suggesting new therapeutic approaches in IBD [3]. 

While all patients with IBD suffer from an increased risk for developing CAC, it was shown that the duration and anatomical extent of disease are well-established risk factors for cancer development. Correspondingly, patients with a course of disease longer than 10 years or pancolitis are at an increased risk [4]. Therefore, most guidelines recommend surveillance colonoscopy as the gold standard for diagnosis of intraepithelial neoplasia and cancer in IBD [4].

Nevertheless, to date, no randomized controlled study could show a reduced risk of CAC development by surveillance colonoscopy in IBD [5]. In this context, one retrospective case-control study assessed the severity of inflammation as a risk factor for CAC in UC and found that inflammation measured by endoscopy, which was significant at univariate analysis, was not a significant determinant of cancer risk in a multivariate model [6]. Only the degree of inflammation over time as assessed by histopathology was considered to be an independent risk factor for CAC development [6]. This finding was confirmed by Mathy and coworkers who demonstrated that histologic, rather than endoscopic assessment of inflammation, serve as a better determinant for CAC risk [7]. Although CAC may occur in elevated protruded lesions, it may also develop in normal-appearing (i.e., “flat”) mucosa. Data from the literature indicate that in 50%–80% of cases with colitis-associated neoplasms the lesions are not visible upon endoscopy [8]. This aspect limits the usefulness of colonoscopic visualization and mandates the need for multiple random biopsies, which is a time-consuming, costly, and ineffective endeavour.

Thus it becomes obvious that there is a need for new and more advanced endoscopic imaging techniques for surveillance in IBD. In recent years, new emerging endoscopic imaging techniques were introduced, allowing a detailed analysis of more mucosal and submucosal details. This paper describes the concept of advanced endoscopic imaging for the diagnosis and characterization of Crohn's disease.

## 2. Advanced Endoscopic Imaging Methods

### 2.1. Magnification Endoscopy and Dye-Based Chromoendoscopy

**Figure 1:**
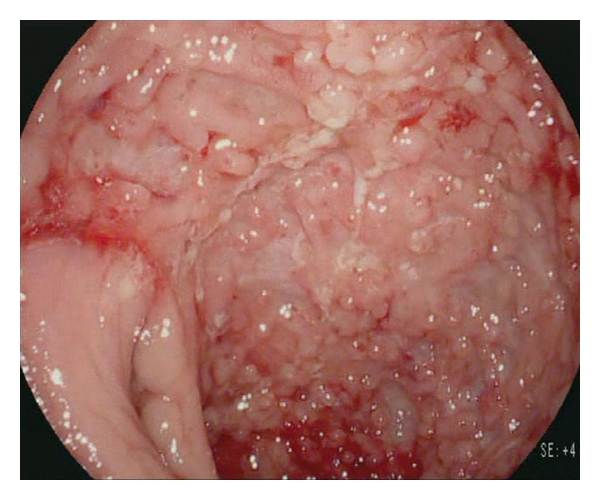
High-definition white-light endoscopy of severe Crohn's disease with multiple longitudinal ulcers and spontaneous bleeding of the inflamed mucosa.

**Figure 2:**
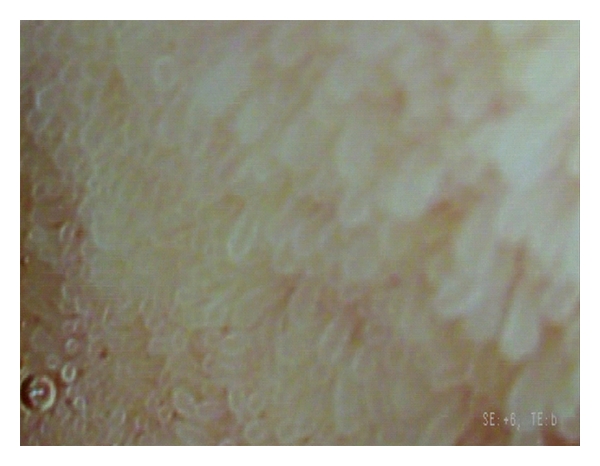
High-magnification endoscopy using i-scan with surface enhancement and tone enhancement clearly visualizes individual intestinal villi with normal shape and size in a patient with quiescent Crohn's disease.

Magnification endoscopy utilizes a movable lens to vary the degree of magnification up to 150-fold, thereby allowing to characterize the mucosal surface (e.g., pit pattern classification of colon polyps as suggested by Kudo et al. in 1996) [9] (Figures 1 and 2).

Chromoendoscopy is divided into dye-based chromoendoscopy (DBC) and dye-less chromoendoscopy (DLC). DLC is further comprised of optical chromoendoscopy and virtual chromoendoscopy. The basic principle of chromoendoscopy is to enhance the mucosal detail and/or submucosal vascular network with the use of various dyes or endoscopic/optical and computer-based color programs. The contrast enhancement of the mucosal surface often results in an improved detection of subtle lesions. DBC uses different dye agents which are divided into absorptive agents (Lugol, methylene blue, toluidine blue, and cresyl violet), contrast agents (indigo carmine, acetic acid) and reactive staining agents (congo red, phenol red). Dye agents are mostly applied via standard spraying or plain biliary ERCP catheters [10]. Various studies have shown the potential of chromoendoscopy to enhance detection of preneoplastic and neoplastic lesions in IBD. One randomized, controlled trial evaluated whether chromoendoscopy using 0.1% methylene blue might facilitate early detection of intraepithelial neoplasia and CAC in UC. Chromoendoscopy permitted more accurate diagnosis of extent and severity of inflammatory activity in UC compared with conventional colonoscopy and improved significantly the early detection of intraepithelial neoplasia and CAC in patients with UC [11]. Another back-to-back colonoscopy study evaluated pancolonic chromoendoscopy with 0.1% indigo carmine for detection of dysplasia in UC. Nontargeted biopsies detected no dysplasia in 2904 biopsies, while the targeted biopsy protocol with pancolonic chromoendoscopy required fewer biopsies (157) and detected nine dysplastic lesions [12]. These results were confirmed in a large prospective trial including 102 patients with chronic colitis using chromoendoscopy with methylene blue. Targeted biopsies with dye spray revealed significantly more dysplasia than random biopsies and more than the targeted nondye spray protocol [13]. A meta-analysis of six randomized controlled trials evaluated the diagnostic accuracy of chromoendoscopy for dysplasia detection in UC. The meta-analysis demonstrated a pooled sensitivity of 83.3%, specificity of 91.3%, and diagnostic odds ratio of 17.5. It was concluded that chromoendoscopy has a medium-to-high sensitivity and a high diagnostic accuracy for detection of dysplastic lesions in UC [14].

Typical endoscopic aspects of conventional endoscopy and chromoendoscopy were recently summarized by the group from the Hyogo College of Medicine in Japan [15].

Hurlstone and coworkers analyzed high-magnification chromoscopic colonoscopy in UC as a tool for reliable assessment of disease extent [16]. 325 patients were prospectively included, and magnification endoscopy was significantly better than conventional colonoscopy for predicting disease extent *in vivo*. The same group prospectively analyzed indigo carmine-assisted high-magnification chromoscopic colonoscopy for the detection and characterisation of intraepithelial neoplasia in UC. Significantly more intraepithelial neoplastic lesions were detected in the magnification chromoendoscopy group compared with controls. Moreover, chromoendoscopy increased the number of flat lesions with intraepithelial neoplasia detected compared with controls [17]. Another group studied the pit patterns in rectal mucosa by magnifying colonoscopy as a marker of relapse in patients with quiescent UC [18]. Multivariate proportional hazard model analysis showed that pit pattern analysis using magnification endoscopy could significantly predict relapse of disease in patients with quiescent ulcerative colitis. These results were also confirmed by Watanabe and coworkers [19].

- Pancolonic chromoendoscopy and targeted biopsies of suspicious lesions represent a more effective surveillance method in IBD than taking only multiple nontargeted biopsies.
- Magnification chromoendoscopy improves the detection of preneoplastic and neoplastic mucosal changes.
- Magnification endoscopy has the potential to predict relapse in patients with quiescent disease.

### 2.2. Dye-Less Chromoendoscopy

**Figure 3:**
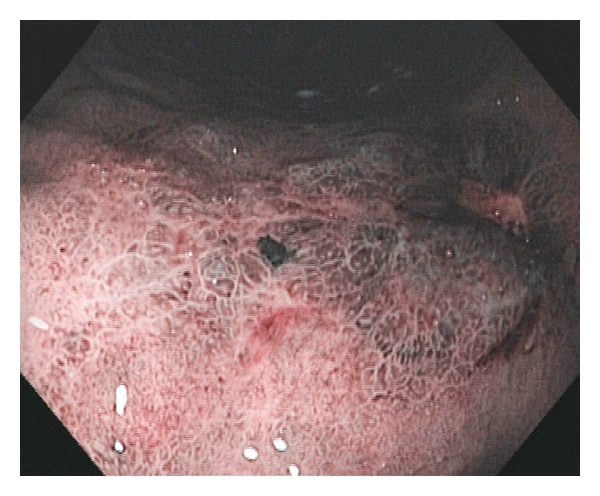
Large ulcer examined with narrow band imaging. NBI visualizes irregular surface architecture and disturbed vascular structures. Histopathology confirmed diagnosis of cancer.

**Figure 4:**
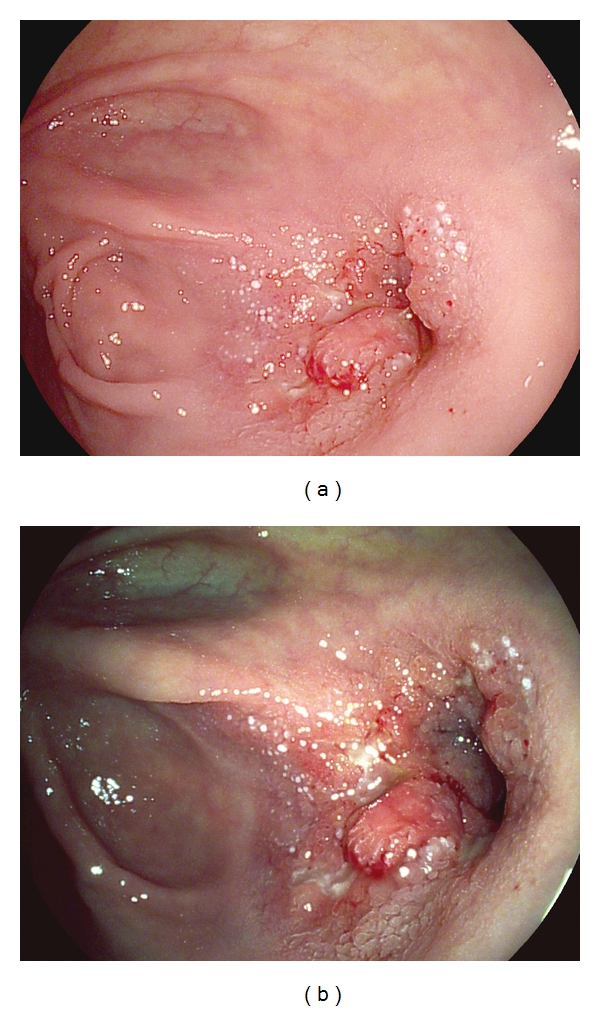
(a) highlights high-definition white-light endoscopic picture of anastomotic stricture in a patient suffering from Crohn's disease. (b) shows virtual chromoendoscopy with i-scan to better characterize the stricture. i-scan revealed enhanced vasculature and fibrinous lesions consisting with an active inflammation.

DLC is divided into optical chromoendoscopy including narrow band imaging (NBI; Olympus, Tokyo, Japan Figure 3) and virtual chromoendoscopy including i-scan (Pentax, Tokyo, Japan Figure 4) and Fujinon intelligent color enhancement (FICE; Fujinon, Tokyo, Japan). While NBI is based on optical filters within the light source of the endoscope which narrow the bandwidth of spectral transmittance, thereby enhancing blood vessels, i-scan and FICE use digital postprocessing for computed spectral estimation for better tissue contrast [20].

Various studies have addressed the potential of NBI for diagnosis and characterization of preneoplastic and neoplastic changes in IBD. One recent published article reviews endoscopic findings under narrow band imaging. It was shown that NBI findings strongly correlated with histologic findings including crypt distortion, goblet cell depletion, and basal plasmacytosis, therefore, harbouring the potential to better assess histologic severity in only mild and inactive disease [21]. Very recently, van den Broek and coworkers performed a randomized crossover trial in which patients with UC underwent both NBI and high-definition (HD) white-light colonoscopy in a randomized order. It was shown that NBI does not improve the detection of neoplasia in patients with UC compared to HD endoscopy. In addition, NBI proved unsatisfactory for differentiating neoplastic from nonneoplastic mucosa [22]. The same group assessed the value of endoscopic trimodal imaging for surveillance in UC. Fifty patients underwent surveillance colonoscopy and each colonic segment was inspected twice, once with autofluorescence imaging (AFI) and once with white-light endoscopy, in random order. All detected lesions were inspected by NBI for Kudo pit pattern analysis and additional random biopsies were taken. AFI improved the detection of neoplasia and decreased the yield of random biopsies. Pit pattern analysis by NBI has a moderate accuracy for the prediction of histology, whereas AFI colour appears valuable in excluding the presence of neoplasia [23]. Kudo et al. analyzed the mucosal vascular pattern (MVP) in UC using both conventional and NBI colonoscopy. In this study, NBI colonoscopy was more precise to determine the grade of inflammation in patients with quiescent UC compared to histology as the gold standard [24]. In another study, the same group performed a pilot study of magnification colonoscopy with NBI for diagnosis of dysplasia in UC. The surface pattern was determined to be either honeycomblike, villous, or tortuouslike. By taking the surface pattern into account, the rate of positive dysplasia was higher in the tortuous pattern than in the honeycomblike or villous pattern group. Therefore, the tortuous pattern determined by NBI colonoscopy may be a clue for the identification of dysplasia during surveillance for UC [21]. Very recently it was also shown that NBI appears to be a less timeconsuming and equally effective alternative to chromoendoscopy for the detection of intraepithelial neoplasia. NBI resulted in a significantly inferior false-positive biopsy rate and a similar true-positive rate. However, given the NBI lesion and patient miss rates, NBI could not be recommended as the standard surveillance technique in IBD [25]. Another pilot study addressed the question if NBI could successfully assess mucosal angiogenesis in IBD. In areas that were endoscopically normal but positive on NBI, there was a significant increase in mucosal angiogenesis. Therefore, NBI may allow *in vivo* imaging of intestinal neoangiogenesis in IBD patients [26].

Only limited data are currently available on virtual chromoendoscopy techniques in IBD. Up to now, only one study evaluated FICE in the IBD setting, showing that the system could not improve the detection or delineation of ulcers and erosions in CD [27]. Another study compared chromoendoscopy with methylene blue (0.1%) and HD colonoscopy using i-scan. HD colonoscopy with and without i-scan was feasible to unmask a plethora of small lesions but chromoendoscopy could even increase the number. Moreover, i-scan was able to predict neoplasia as precisely as chromoendoscopy [28]. A randomized controlled study evaluated HD colonoscopy in conjunction with i-scan or standard video colonoscopy for the detection of colorectal lesions. HD endoscopy with i-scan detected significantly more patients with colorectal neoplasia (38%) compared with standard resolution endoscopy (13%). Significantly more neoplastic lesions and more flat adenomas could also be detected using HD endoscopy with i-scan [29]. Recently, our group successfully studied the impact of i-scan for prediction of mucosal inflammation in IBD. Patients underwent total colonoscopy and were examined using both HD (Group A) and HD plus i-scan colonoscopy (Group B). Agreement between endoscopic prediction of disease severity and histologic findings was 65% in group A and 88% in group B. When comparing the endoscopic prediction of extent of inflammatory activity with the histologic results from the corresponding specimens, we found agreement in 42% in group A and 85% in group B. Compared with histological results of inflammation, i-scan significantly enabled more precise diagnosis of mucosal inflammation compared to conventional colonoscopy [30].

- Dye-less chromoendoscopy offers the potential to replace conventional dye-based chromoendoscopy for lesion detection and assessment of disease severity in IBD.
- Virtual chromoendoscopy with i-scan is superior to standard video colonoscopy for the detection of colorectal neoplasia.
- i-scan significantly improves the diagnosis of severity and extent of mucosal inflammation in patients with IBD.

### 2.3. Balloon-Assisted Enteroscopy

Although standard small-bowel endoscopy plays a pivotal role in the management of patients with IBD, its location in the diagnosis and treatment algorithm is still not clearly defined [31]. The term “balloon-assisted enteroscopy” (BAE) was first proposed by Mönkemüller and coworkers and summarizes three different endoscopic methods for small-bowel imaging: (1) double-balloon enteroscopy (DBE; Fujinon, Tokyo, Japan); (2) single-balloon endoscopy (SBE; Olympus, Tokyo, Japan); (3) NaviAid (Pentax, Tokyo, Japan). All systems allow deep intubation of the small bowel for diagnostic and therapeutic interventions (e.g., for hemostasis, dilation, or biopsy acquisition). Combination of oral and anal insertion routes are used is to achieve complete examination of the small bowel [32].

Up to now few studies have reported a 30–48% diagnostic yield of DBE when evaluating patients with suspected Crohn's disease [32–36]. Complications are rare and occur in <1% of cases while balloon dilation of strictures has a reported perforation risk of up to 3% [34]. Nevertheless, BAE should not be considered as the first-line procedure in the evaluation of suspected small-bowel CD, as the procedure is somehow invasive, time- and cost- expensive, and mostly limited to specialized centers. Therefore, in patients with suspected small-bowel CD without strictures or stenosis capsule endoscopy should firstly be considered [31]. However, BAE allows for the retrieval of tissue for analysis and permits therapeutic interventions such as stricture dilation.

### 2.4. Capsule Endoscopy

Capsule endoscopy (CE) was introduced in 2002. Currently, various capsule systems from different companies are available for examination of the esophagus, small bowel, and colon [36]. The capsule movement is passively propelled through the intestine by peristalsis while transmitting color images of the intestine [37]. CE offers the theoretical advantage of visualizing the whole small bowel. Nevertheless, the caecum can not be reached in about 8%–40% of cases [31]. Multiple studies have shown the impact of CE to diagnose CD-associated changes like ulcers, erosions, erythema, aphthae, and strictures [38–40]. In the study by Dubcenco et al. CE yielded a sensitivity and specificity of 89.6% and 100%, respectively, and a positive predictive value and negative predictive value of 100% and 76.9%, respectively, for diagnosis of active small-bowel CD [39]. However, none of these studies used unequivocal gold standards and the diagnosis of Crohn's was always supported by the clinical presentation. Thus, at present diagnosis of Crohn's disease cannot be solely based on CE examination.

- In case of suspected small bowel, crohn's disease without strictures or stenosis capsule endoscopy should be preferred.
- Balloon-assisted enteroscopy offers the potential to perform interventions within the small bowel and is associated with a low complication rate.

### 2.5. Confocal Laser Endomicroscopy

**Figure 5:**
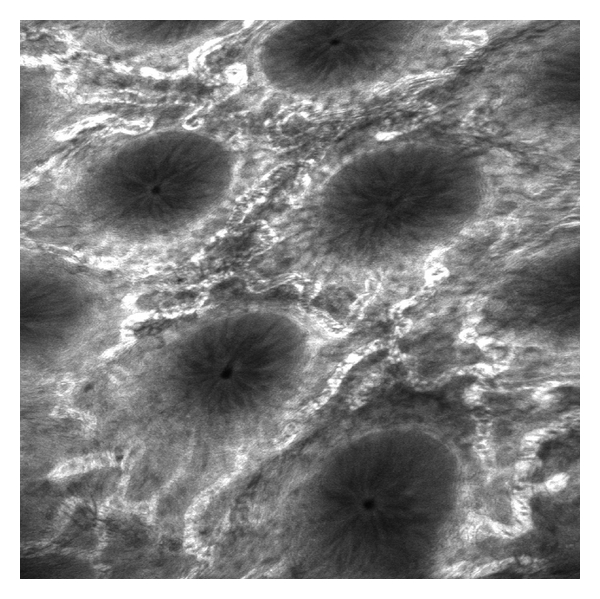
Confocal laser endomicroscopy reveals normal appearing microvessels within the lamina propria and colonic crypts regular in size and shape in patient with quiescent Crohn's disease.

Confocal laser endomicroscopy (CLE) was introduced in 2004 and has rapidly emerged as a promising approach to obtain real time *in vivo* histology during ongoing endoscopy (Figure 5). The technique is based on tissue illumination with a blue laser light after topical or systemic application of fluorescence agents. The most commonly used fluorescence agent is fluorescein sodium which is administered intravenously thereby providing systemic tissue staining. Currently, two FDA-approved and *CE*-certified devices are available. One is integrated into the distal tip of a high-resolution endoscope (“integrated”, iCLE; Pentax, Tokyo, Japan) and one represents a stand-alone confocal probe which is capable of passing through the working channel of most standard endoscopes (“probe-based”, pCLE; Cellvizio, Mauna Kea Technologies, Paris, France) [41].

Kiesslich and coworkers impressively demonstrated that combination of chromoendoscopy (methylene blue; 0.1%) and endomicroscopy could detect 4.75-fold more neoplasias in surveillance colonoscopy of patients with UC compared to conventional endoscopy. Moreover, 50% less biopsy specimens were required and CLE could predict neoplastic changes with a sensitivity, specificity, and accuracy of about 95%, 98%, and 98%, respectively [42]. Also CLE was feasible to differentiate dysplasia-associated lesion or mass (DALM) and sporadic adenoma (adenoma-like mass; ALM) with a high accuracy of 97% and an excellent agreement between CLE and histological diagnosis (*κ* = 0.91) [43]. 

One study by Li and coworkers proposed a classification system of inflammatory activity in IBD based on a 4-grade classification system of crypt architecture, as well as by analysis of microvascular alterations and fluorescein leakage. Both, assessment of crypt architecture and fluorescein leakage with CLE showed good correlations with histological results. Moreover, CLE seemed to be more accurate than conventional white-light endoscopy for evaluating macroscopically normal appearing mucosa. More than half of the patients with normal mucosa seen on conventional white-light endoscopy showed acute histologic inflammation, whereas no patients with normal mucosa or with chronic inflammation seen on CLE showed acute inflammation on histology [44].

Our group evaluated endomicroscopy for *in vivo* diagnosis of Crohn's-disease-associated histological changes. Endomicroscopy was able to diagnose Crohn's-disease-associated changes with high accuracy. Furthermore, endomicroscopy could detect residual macroscopic nonvisible mucosal inflammation as precisely as histology (kappa values 0.8) [45].

- CLE can detect more neoplasms in surveillance colonoscopy of patients with IBD and could predict neoplastic changes with high accuracy.
- CLE can reliably predict inflammatory activity in IBD during ongoing endoscopy, even in patients with macroscopically uneventful mucosa.

### 2.6. Endocytoscopy

**Figure 6:**
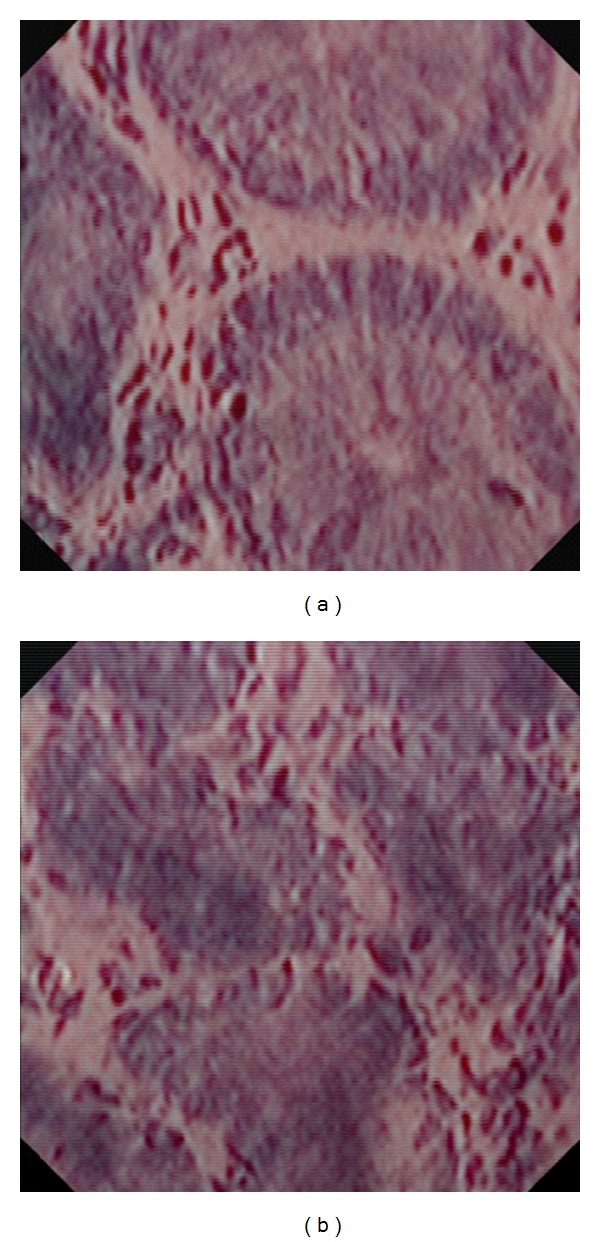
Endocytoscopy with 1390-fold magnification in quiescent (a) and active (b) Crohn's disease. Endocytoscopy enables clear visualization of different cellular structures within the intestinal mucosa, including the size, arrangement, and density of cells.

Endocytoscopy (EC Figure 6) enables *in vivo* microscopic imaging at a magnification up to 1390-fold magnification, thereby allowing the analysis of mucosal structures at the cellular level. EC is based on the principle of contact light microscopy and therefore only allows visualisation of the very superficial mucosal layer [46]. The technique was shown to be reliable for the examination of mucosal surfaces [47, 48]. In addition, EC could predict neoplasia in aberrant crypt foci and could distinguish neoplastic from nonneoplastic colorectal lesions [49, 50]. Our group assessed the value of endocytoscopy to determine single inflammatory cells during ongoing endoscopy in patients with IBD. EC enabled clear visualization of different cellular structures within the intestinal mucosa, including size, arrangement, and density of cells. Furthermore, size and shape of nuclei and the nucleus-to-cytoplasm ratio were visualized. According to these changes, it was possible to distinguish in real-time neutrophilic granulocytes, basophilic granulocytes, eosinophilic granulocytes, and lymphocytes by EC [51].

- EC harbours the potential to accurately determine various inflammatory mucosal cells during ongoing endoscopy in IBD and thus the severity of the inflammation.

## 3. Conclusion

Advanced endoscopic imaging in IBD has seen a major innovation revolution during the last 10 years, including both vital and virtual chromoendoscopy. By using capsule endoscopy or balloon-assisted enteroscopy, the endoscopist is now able to evaluate the entire small bowel and to perform endoscopic therapies at a place* where no one has gone before*. Moreover, recent advances in endoscopic imaging now enable the endoscopist to obtain real time *in vivo* histology during ongoing endoscopy by using endomicroscopy or endocytoscopy. These emerging imaging modalities enable the endoscopist to detect and characterize more preneoplastic and neoplastic lesions and to predict mucosal inflammation more precisely as compared to conventional white light endoscopy. Recent efforts have been made to introduce molecular imaging in IBD by highlighting inflammatory cells and specific receptors. Results of these trials are highly anticipated and might open new avenues for diagnostic and therapeutic strategies in IBD.
